# Global optical coherence tomography measures for detecting the progression of glaucoma have fundamental flaws

**DOI:** 10.1038/s41433-020-01296-x

**Published:** 2021-01-07

**Authors:** Ashley Sun, Emmanouil Tsamis, Melvi D. Eguia, Jeffrey M. Liebmann, Dana M. Blumberg, Lama A. Al-Aswad, George A. Cioffi, C. Gustavo De Moraes, Donald C. Hood

**Affiliations:** 1 Department of Psychology, Columbia University, New York, NY USA; 2 Einhorn Clinical Research Center, New York Eye and Ear Infirmary of Mount Sinai, New York, NY USA; 3 Bernard and Shirlee Brown Glaucoma Research Laboratory, Department of Ophthalmology, Edward S. Harkness Eye Institute, Columbia University Irving Medical Center, New York, NY USA

**Keywords:** Optic nerve diseases, Tomography

## Abstract

**Objective:**

To understand the problems involved in using global OCT measures for detecting progression in early glaucoma.

**Subjects/Methods:**

Eyes from 76 patients and 28 healthy controls (HC) had a least two OCT scans at least 1 year apart. To determine the 95% confidence intervals (CI), 151 eyes (49 HC and 102 patients) had at least two scans within 6 months. All eyes had 24-2 mean deviation ≥-6dB. The average (global) thicknesses of the circumpapillary retinal nerve fibre layer (cRNFL), G_ONH_, and of the retinal ganglion cell layer plus inner plexiform layer (RGCLP), G_mac_, were calculated. Using quantile regression, the 95% CI intervals were determined. Eyes outside the CIs were classified as “progressors.” For a reference standard (RS), four experts evaluated OCT and VF information.

**Results:**

Compared to the RS, 31 of the 76 (40.8%) patient eyes were identified as progressors (RS-P), and 45 patient, and all 28 HC, eyes as nonprogressors (RS-NP). The metrics missed (false negative, FN) 15 (48%) (G_ONH_) and 9 (29%) (G_mac_) of the 31 RS-P. Further, G_ONH_ and/or G_mac_ falsely identified (false positive, FP) 10 (22.2%) of 45 patient RS-NP eyes and 7 (25%) of the 28 HC eyes as progressing. Post-hoc analysis identified three reasons (segmentation, centring, and local damage) for these errors.

**Conclusions:**

Global metrics lead to FPs and FNs because of problems inherent in OCT scanning (segmentation and centring), and to FNs because they can miss local damage. These problems are difficult, if not impossible, to correct, and raise concerns about the advisability of using G_ONH_ and G_mac_ for detecting progression.

## Introduction

Detecting the progression of glaucoma is a challenge for the clinician. Traditionally, the most commonly used quantitative techniques involved the mean deviation (MD) of the 24-2 visual field (VF), obtained with standard automated perimetry. With the advent of optical coherence tomography (OCT), the average thickness of the circumpapillary retinal nerve fibre layer (cRNFL) became a common measure of progression. This measure, called global cRNFL thickness, has been incorporated into commercial OCT reports. With the recent incorporation of OCT scanning of the macula, an average (global) measure of the retinal ganglion cell plus inner plexiform layer (RGCLP) thickness also has been employed to track progression, and a number of studies have compared these two OCT global measures [1–7].

However, these two measures, global cRNFL (G_ONH_) and global RGCLP (G_mac_), miss early glaucomatous damage clearly visible on probability/deviation maps, which display abnormal regions of RNFL and/or RGCLP thickness [8–10]. Thus, it is likely that these two measures will also miss clear progression of glaucoma, while also falsely identifying some eyes as progressors.

Our purpose here was to understand the problems involved in using global OCT measures for detecting progression in early glaucoma. First, we show, as expected, that the conventional thickness measures, G_ONH_ and G_mac_, combined with a traditional event-based analysis, lead to both excessive false positives (FPs) and false negatives (FNs). Second, and most importantly, we identify the reasons for these errors via a post-hoc analysis.

## Methods

### Participants

There were 104 study eyes from 104 individuals; 76 were from glaucoma or glaucoma suspect patients. The remaining 28 eyes were healthy controls (HCs) with normal fundus examination, normal VFs, and IOP < 22 mmHg. All eyes had 24-2 MD better than -6 dB and at least two OCT scans: a baseline scan and a scan obtained at least 1 year after the baseline (mean: 24.9 ± 8.7 months, range 12–42 months). All individuals were enroled in Columbia University’s prospective study, Macular Damage in Early Glaucoma and Progression (ClinicalTrials.gov: NCT02547740).

Study procedures followed the tenets of the Declaration of Helsinki and Health Insurance Portability and Accountability Act and were approved by the Institutional Review Board of Columbia University. Written informed consent was obtained from all participants.

### OCT data

**Fig. 1:**
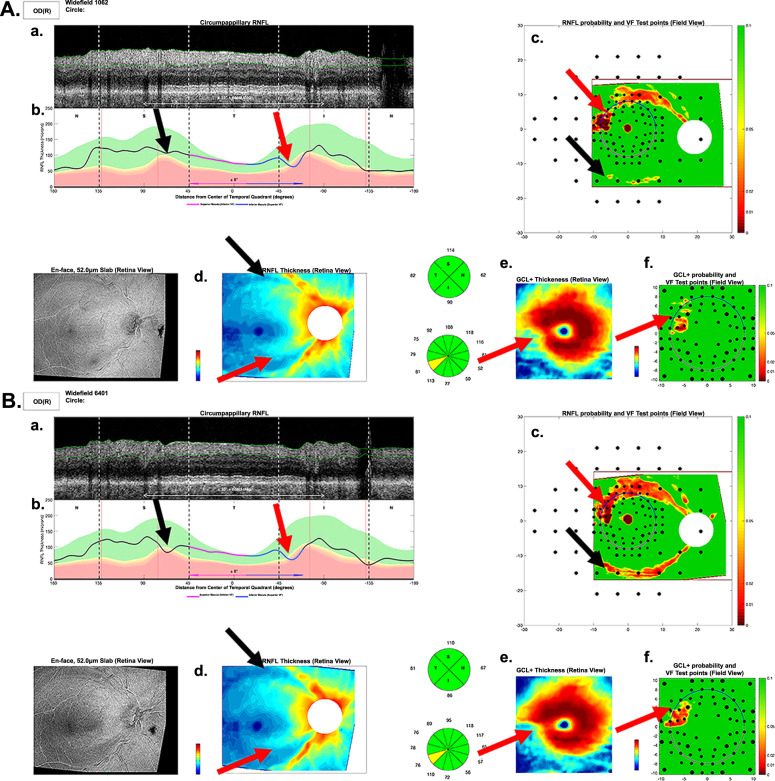
Local defect. Example of an eye in the likely progression reference standard (RS-P) that showed “statistical progression” according to the global RGC (G_mac_) metric, but not according to the global RNFL (G_ONH_) metric. The arrows indicate damage in the baseline (**A**) and follow-up (**B**) reports, which was not detected by the G_ONH_ metric. The red and black arrows indicate inferior and superior damage respectively, both of which are subtle local arcuate regions showing progression. Panels a–f show essential parts of the one-page report: Derived circle b-scan (a) and its corresponding cRNFL thickness plot (b); RNFL (d) and RGC (e) thickness maps; RNFL (c) and RGC (f) probability/deviation maps.

Widefield (12 × 9 mm) swept-source OCT volume scans (Atlantis; Topcon Inc., Tokyo, Japan) were obtained for each eye. Every scan was rotated to a common fovea-to-disc angle, which accounted for head-eye torsion, and to some extent anatomical differences, as previously described [11], and currently incorporated in a commercial report similar to the one in Fig. 1 generated by our custom programme. A derived B-scan image (Fig. 1Aa) was generated from the widefield scan for a circle 3.45-mm in diameter centred on the optic disc. The cRNFL thicknesses were measured (black-magenta-blue-black curve in Fig. 1Ab). A RNFL thickness map (Fig. 1Ad) was obtained from the widefield scan. A portion of the widefield scan, 6 × 6 mm centred on the fovea, was used to produce a RGCLP thickness map (Fig. 1Ae). Age-corrected RNFL (Fig. 1Ac) and RGCLP (Fig. 1Af) probability maps were created based on these thickness maps and normative controls [12].

### Establishing progression with OCT summary metrics

Global cRNFL (G_ONH_) and global RGCLP (G_mac_) average thicknesses were calculated for each eye at each visit. The thresholds [95% confidence interval (CI)] to identify statistically significant event-based progression in the study group were derived from a short-term group after performing quantile regression [13], which is analogous to how event-based progression is defined with commercially available VFs and OCT. Details of this event-based methodology are provided in the Supplementary Information (Supplementary Fig. 1).

These 95% thresholds were then applied to the 104 eyes of the study group. Eyes whose G_ONH_ or G_mac_ metric on the follow-up test were equal or greater than the 95% CI were classified as “statistical progressors”.

#### Reference standard (RS) for progression

Our objective here was to identify factors affecting changes in G_ONH_ or G_mac_ by analysing B-scans (e.g., Fig. 1Aa) and probability maps (e.g., Fig. 1Ac, f) of possible FPs and possible FNs. To identify the eyes that are possible FP and false FN, a reference standard (RS) was used. In particular, four of the authors independently decided on progression or no progression after evaluating all available OCT and VF tests, and all OCT reports with probability maps (Fig. 1). For the 104 study eyes, the average number of visits was 8.3 ± 2.6. Initially, the experts agreed for 98 eyes, and consensus was reached for the remaining 6 after they reviewed the cases together.

## Results

### Progressors according to metrics

**Fig. 2:**
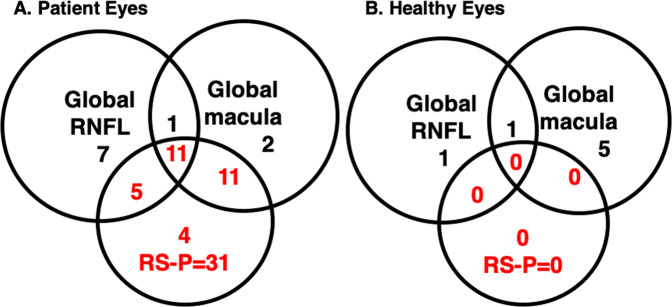
Comparison of the performance of the global RNFL (G_ONH_) and global RGCL (G_mac_) metrics in identifying progression, against progressors in the clinical reference standard (RS-P). The eyes are split into patient (**A**) and healthy (**B**) eyes.

The two global summary metrics (i.e., G_ONH_ and G_mac_) identified a similar number of patient eyes as ‘statistical progressors’; 24 for G_ONH_ and 25 for G_mac_ (Fig. 2A). About half, 12 eyes, were ‘statistical progressors’ according to both metrics.

The G_ONH_ and/or G_mac_ metric also identified 7 of the 28 (25%) HC eyes as “statistical progressors” (Fig. 2B). These seven eyes were clearly FP as they were HCs with no signs of glaucomatous damage. Of these seven FP, two were FP on G_ONH_ and six on G_mac_, and one on both.

### Comparison of clinical RS and summary metrics

Based upon the RS, 31 of the 76 (40.8%) patient eyes showed signs of progression (RS-P), while none of the 28 HC eyes were identified as RS-P.

#### True positives based upon RS

Of the 31 RS-P eyes, 11 eyes (35.5%) were identified by both metrics as statistical progressors (Fig. 2A). All 11 showed clear signs of progressing damage on both the RNFL and RGCLP thickness and probability maps. An example of a true positive for both metrics is provided in the Supplementary Information (Supplementary Fig. 2/Supplementary Video 1).

#### FNs based upon RS

**Fig. 3:**
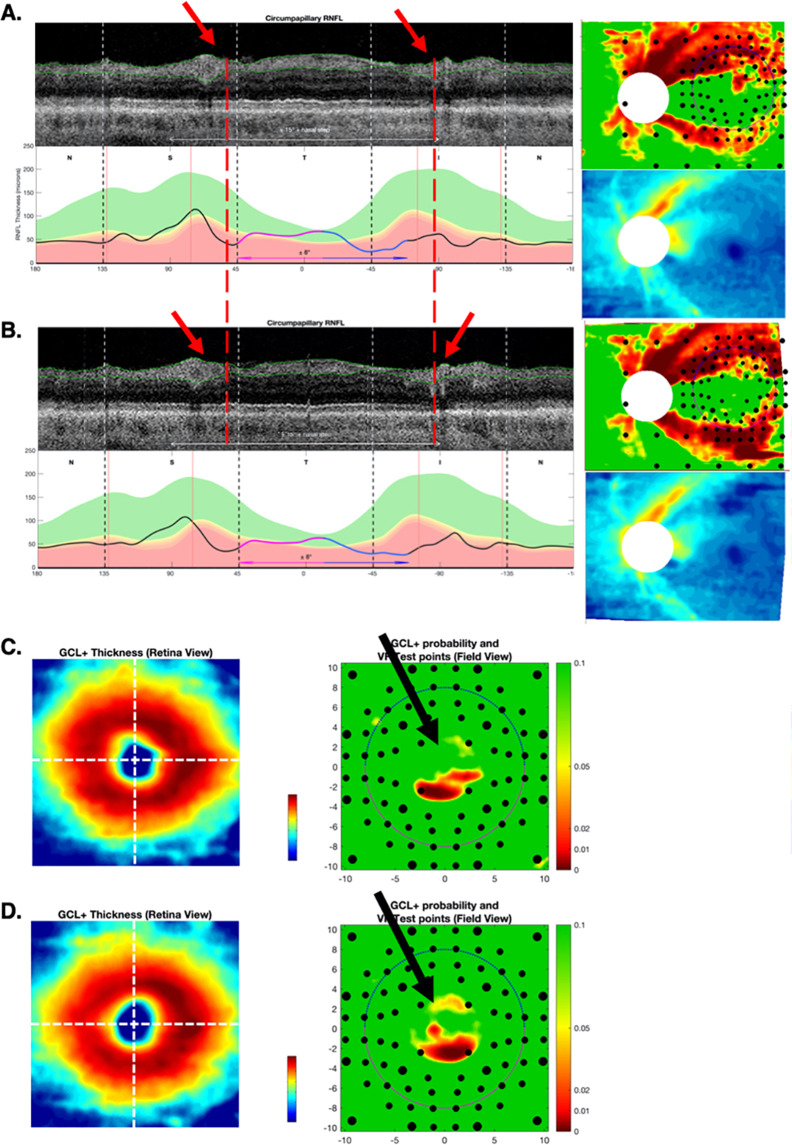
Differences in widefield centring. Example of an eye with different disc centring between baseline (**A**) and follow-up (**B**) scans. The red arrows and dashed red lines confirm the misalignment by the position of the blood vessel shadows on the circle scan. A second example shows different foveal centreing (white crossed lines) in the baseline (**C**) and follow-up (**D**) RGC+ probability plots. The black arrows indicate areas with subtle differences in the probability values due to foveal centring rather than true glaucomatous progression.

Only four (12.9%) RS-P eyes were missed by both metrics (Fig. 3A). All four showed clear glaucomatous damage when the entire report was evaluated.

The fact that only four eyes were missed by both G_ONH_ and G_mac_ underestimates the extent of the problem with the clinical use of these metrics. Suppose we were to use “abnormal on G_ONH_ OR G_mac_” for clinical decision making. Then, although the FN rate for RS-P would be 12.9% (4 eyes), the FP rate for the HC would be 25% (Table 1). Thus, we need to understand the FNs for G_ONH_ and G_mac_ alone. A total of 20 (64.5%) of 31 RS eyes were missed by one or both metrics. That is, in addition to the 4 missed by both, 16 other eyes were missed by either G_ONH_ or G_mac_.

**Table 1:** Percent/(number) of false negatives (FN) and false positives (FP) based on global retinal nerve fibre layer (G_ONH_) and global retinal ganglion cell plus inner plexiform layer (G_mac_) metrics.

	G_ONH_	G_mac_	OR	AND	G_ONH_ 5 μm
FN (*n* = 31)	48.3% (15)	29.0% (9)	12.9% (4)	64.5% (20)	67.7% (21)
FP (*n* = 45)	17.8% (8)	6.7% (3)	22.2% (10)	2.2% (1)	8.9% (4)
Accuracy (*n* = 76)	69.7% (23)	84.2% (12)	81.6% (14)	72.4% (21)	67.1% (25)
FP (*n* = 28 HC)	7.1% (2)	21.4% (6)	25% (7)	7.1% (2)	0% (0)

##### Missed only by G_mac_

Five (5) of the 31 eyes categorised as RS-P were identified as ‘statistical progressors’ on the G_ONH_, but not G_mac_, metric. Three of the five eyes showed clear thinning on the RGCLP thickness map, even though the G_mac_ metric failed to identify the eye as a progressor.

##### Missed only by G_ONH_

Eleven (11) of the 31 eyes in the RS were identified as ‘possible progressors’ on the G_mac_, but not the G_ONH_, metric. Seven of these 11 G_ONH_ FN eyes showed clear progressive thinning on the RNFL, which was not detected by the G_ONH_ metric. Figure 1 shows the reports for one of these eyes. The arrows point to corresponding regions with clear progression in the inferior retina and disc (red) and the superior retina and disc (black).

Three additional examples are provided in the Supplementary Information where the metrics failed to detect the RS-P eyes correctly (Results). One eye was missed by both metrics (Supplementary Fig. 3/Supplementary Video 2), while another only by G_ONH_ (Supplementary Fig. 4/Supplementary Video 3), and the last only by G_mac_ (Supplementary Fig. 5/Supplementary Video 4).

#### FPs based upon RS-NP

First, of the 28 HC eyes, G_ONH_ and G_mac_ falsely classified 2 (G_ONH_) and 6 (G_mac_) eyes as statistical progressors. Further, of the 45 patient eyes judged to be RS-NP, 8 (G_ONH_) and 3 (G_mac_) eyes were classified as “statistical progressors”, with 1 eye judged as progressing by both.

### Post-hoc analysis of FP and FN

A post-hoc analysis was performed to understand the possible reasons for the disagreement between the metrics and the RS. This analysis identified three possible reasons: (1) local damage; (2) disc and fovea centring; and (3) segmentation errors.

#### Local damage

Of the 20 FN eyes missed by one or both metrics, 6 had local defects (2 FNs on both metrics, 3 on G_ONH_, and 1 on G_mac_). The reports (panels A and B in Fig. 1) are for an eye “progressing” according to the G_mac,_ but not the G_ONH_. Local defects in both the superior (black arrows) and inferior (red arrows) retina deepen over time. The G_ONH_ metric missed this local damage.

#### Differences in centring of derived circle or fovea

In six of the eyes where the G_ONH_ metric disagreed with the RS (four FN, two FP), there was a small difference in centring of the optic disc between days identified on the reports. Figure 3A, B shows an example where the disc was centred differently on the two reports. This resulted in a change in the location of the derived circle scan, as can be seen by the shadows of the blood vessels (red arrows and dashed lines). This resulted in an FN for G_ONH_. For these six eyes, the change in G_ONH_ was small (average of 3.8 μm), only just outside the 95% CI. (Overall, based upon the quantile regression, the 95% CI for G_ONH_ ranged from 3.2 to 3.6 μm.) Note that in five of these six eyes, G_mac_, which does not depend upon disc centring, agreed with the RS-P.

A similar problem can occur via small differences in the centring of the fovea for the G_mac_ analysis. This appeared to be the primary reason for five HC eyes that were FP only on the G_mac_. For example, in Fig. 3C, D, the ring-like artefact in the RGCLP probability plot (known to be due to anatomical differences of the fovea) suggests a small difference in centreing [14]. For the five eyes, the G_mac_ change ranged from only 1.4 to 1.5 μm, a large value relative to the 95% CI which ranged from 1.0 to 1.4 μm. Note, the foveal centring should only affect the G_mac_. Consistent with this, G_ONH_ agreed with the RS for all five eyes.

#### Segmentation errors

**Fig. 4:**
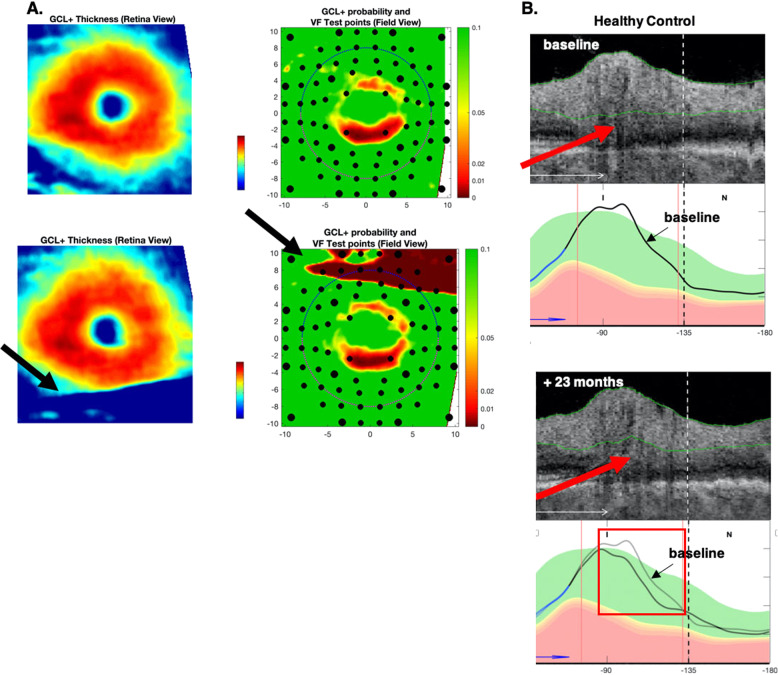
Segmentation errors. Example of a scanning artefact in the macula (**A**), with the baseline RGC+ scans in the bottom row showing scan artefact (black arrows). The artefact is indicated by the black arrows. Example of a segmentation error around blood vessels (**B**), indicated by the red arrows.

Segmentation errors can affect the metrics. Figure 4A shows an example where the segmentation, secondary to a scanning artefact, clearly affected the G_mac_ value. While large errors such as this were rare, more subtle segmentation errors undoubtedly occurred and would be harder to detect. Figure 4B shows an example where a subtle segmentation error (red arrows) resulted in a decrease in the cRNFL thickness in the follow-up scan of this HC eye. The G_ONH_ value changed by 4.6 μm, resulting in a FP, as the 95% CI was 3.1 μm. By superimposing the cRNFL plots for the two scan dates (lower right panel), we estimate that this segmentation error contributed about 4 μm to the change in G_ONH_. Thus, small segmentation errors can lead to FP or FN errors.

## Discussion

We evaluated the performance of two common metrics used for detecting progression of glaucoma, global cRNFL thickness (G_ONH_) and global RGCLP thickness (G_mac_). Consistent with previous studies, these metrics identified a similar number of eyes with a standard event-based technique [15–17]. In particular, the metrics identified 24 (G_ONH_) and 25 (G_mac_) eyes as “statistical progressors,” with 12 eyes progressing on both. Further, we demonstrated that these conventional thickness measures, combined with a traditional event-based analysis, resulted in both excessive FPs and FNs. A post-hoc analysis uncovered reasons for their poor performance, which was the main purpose of this study.

### An evaluation of metrics

Based upon the RS for the patients, the metrics had relatively high FN and FP rates as shown in Table 1. For example, the eyes showing progression according to our RS-P, the FN rates for G_ONH_ and G_mac_ were 48.4% (15 eyes) and 29.0% (9 eyes) (columns 1 and 2, row 1). Given that only four eyes were missed by both metrics, if we classify an eye as a “progressor” based upon an abnormal G_ONH_ OR an abnormal G_mac_, then the FN rate of 12.9% (column 3, row 1), is considerably lower. However, this OR criterion will increase the FP rate (i.e., decrease specificity). In particular, 10 of the 45 RS-NP eyes would be identified as statistical progressors based upon an abnormal G_ONH_ OR G_mac_, for an FP rate of 22.2% and a specificity of 77.8% (column 3, row 2). Further, 7 of the 28 HC eyes would be identified as statistical progressors, for an FP rate of 25% and a specificity of 75%. Thus, G_ONH_ and G_mac_ metrics are a poor method for detecting progression in this population of eyes with early glaucoma.

### Why are metrics performing poorly?

We identified three reasons why these global metrics perform poorly. First, they can miss local damage. The fact that local damage can be missed is understandable as both metrics are based upon averages of regions larger than these local defects. Second, we found that subtle segmentation errors can produce changes in G_ONH_ and G_mac_ that are large relative to the criterion change used to identify progression. Finally, relatively subtle changes in centring of the fovea or disc can also produce changes in G_ONH_ and G_mac_. As a test of concept, we simulated changes in the centring of the fovea and the disc. According to these simulations, small changes in the centre of the disc can produce a change in G_ONH_ equal to the average 95% CI cutoff. This is consistent with a 2009 study by Cheung et al. [18]. Based upon older time domain OCT circle scans, they estimated that offsets as small as 0.1 mm in disc centring produced on average a change in G_ONH_ of 2.3 μm. Similarly, we found changes in the centre of the fovea as small as 0.5° (about 0.14 mm) can produce a change in G_mac_ equal to or more than the average 95% CI cutoff.

There are two important points to be made about segmentation and centring problems. First, all algorithms make segmentation errors and correcting them is difficult in general, and typically not feasible in a clinical practice [19–21]. Likewise, small changes in centring of disc and/or fovea are difficult to impossible to avoid [22, 23]. Segmentation will affect centring and so will head tilt into the plane of the scan. Currently, there is no way to correct the latter. Second, relatively small changes fall outside the 95% CI for these metrics. In this study, average changes of only 3.4 μm (G_ONH_) and 1.6 μm (G_mac_) are needed. Thus, although the changes in these metrics caused by segmentation and centring are small, they can still lead to both FPs and FNs [18].

Given these three problems, it is not surprising that global metrics are suboptimal for identifying progression. Further, there is no easy fix for these problems. Conventional clinical standards, such as Zeiss’ Glaucoma Progression Analysis (GPA), use longer series (usually at least four tests) in an attempt to overcome some of these issues. Trend- and event-based analysis of a series of tests can potentially reduce the ‘noise’ and exclude outliers, although it is likely that local damage will still be missed, and segmentation and centring errors will still contribute to variability. However, there is a more fundamental problem inherent in the trend-based analysis. We have argued that analyses of long series of tests do not fully answer a crucial clinical question that physicians face in a glaucoma clinic; that is, “has glaucoma progressed since the last visit?” [24].

### Our 95% CI values and the literature

Previous studies using different OCT instruments arrived at a 95% CI near 5 μm for the G_ONH_ metric [25–27]. This lead to the “Rule of 5  μm” used by some clinicians [28]. Some consider changes in G_ONH_ of more than 5 μm as indicating progression. In a longitudinal study, Thompson et al. concluded that a 95% CI of 5 μm resulted in too many FPs due to test-retest variability [28]. Our 95% CI value for G_ONH_ was on average 3.4 μm, smaller than 5 μm. Had we used 5 μm instead, it would have reduced the FP rate, but increased the FN rate, leaving accuracy about the same (Table 1, column 5). The accuracy of these global metrics is poor. Thus, changing cutoffs will only trade off sensitivity vs. specificity; it will not improve accuracy.

### What is the alternative?

We have previously argued that OCT global metrics will miss damage that can be seen on reports such as those in Fig. 1 [12, 29]. As in the case of early detection, we are suggesting that trained observers will outperform G_ONH_ and G_mac_ metrics if they had these reports. Of course, there may be some purposes, such as clinical trials, where qualitative evaluations are not appropriate. For these purposes, we need to find alternatives to global metrics. For detection of glaucoma, we have shown success with an objective structure–function method, as well as a deep learning approach [11, 30–33]. Similar approaches can be applied to progression. For example, the clinician can topographically compare the changes in the VF to the changes in the OCT probability maps, as well as topographically compare the changes in the different OCT maps and images.

### Limitations

There are three limitations to this study worth mentioning. First, the sample is relatively small, although it is hard to see how more eyes will change the fundamental findings here. Second, the design suffers from the general problem facing studies of progression. There is no “gold standard” or “litmus test for progression.” In this study we used an RS based on the consensus of four experts after evaluation of all available structural and functional information. Other progression studies have used, for example, Zeiss’ GPA to confirm the presence of deterioration [34, 35]. Thus, applying different RS will produce different estimates of FP and FN. However, our general conclusions regarding the problems with these metrics should hold. See the Supplementary Figures for proof of concept.

Finally, the eyes in this study were all “early glaucoma,” as defined by 24-2 MD better than -6 dB at baseline. The results here need to be extended to more advanced glaucoma. While it is generally held that one cannot use OCT for eyes with G_ONH_ values less than about 50 μm, we have recently shown this is not true [36].

## Conclusions

Global statistics such as average cRNFL thickness (G_ONH_) and average RGCLP thickness (G_mac_) will miss or overcall progression of glaucoma. There are inherent problems with these methods that will be difficult, if not impossible, to correct. In particular, as they are averages, they can miss local defects. Further, they are prone to FP and FN mistakes due to subtle segmentation and alignment errors of the fovea and disc centres. Approaches are needed which do not rely on these metrics and instead focus on the topographical agreement among the cRNFL, RGCLP, and RNFL thickness measures.

### Summary

#### What was known before

- Average (global) measures of the circumpapillary retinal nerve fibre layer (cRNFL) and the retinal ganglion cell plus inner plexiform layer (RGCLP) thickness are common measures of progression. However, these two measures, global cRNFL (G) and global RGCLP (Gmac), miss early glaucomatous damage. Thus, it is likely that these two measures will also miss clear progression of glaucoma, while also falsely identifying some eyes as progressors.

#### What this study adds

- Global metrics G and Gmac can lead to both false positives and false negatives because of problems inherent in OCT scanning, such as segmentation and centring. In addition, they can miss local damage (false negatives). These problems are difficult, if not impossible, to correct, and raise concerns about the advisability of using global metrics for detecting progression.

## Supplementary information

Supplementary Figure 1: Quantile Regression of Baseline and Follow-Up Visits

Supplementary Figure 2: Example of a true positive for both metrics

Supplementary Figure 3: Example of a false negative for both metrics

Supplementary Figure 4: Example of a false negative for GONH

Supplementary Figure 5: Example of a false negative for Gmac

Supplementary Video 1: True positive for both metrics

Supplementary Video 2: False negative for both metrics

Supplementary Video 3: False negative for GONH only

Supplementary Video 4: False negative for Gmac only

## References

[1] Hou HW, Lin C, Leung CK (2018). Integrating macular ganglion cell inner plexiform layer and parapapillary retinal nerve fiber layer measurements to detect glaucoma progression. Ophthalmology..

[2] Na JH, Sung KR, Baek S (2012). Detection of glaucoma progression by assessment of segmented macular thickness data obtained using spectral domain optical coherence tomography. Investig Ophthalmol Vis Sci.

[3] Iverson SM, Feuer WJ, Shi W, Greenfield DS (2014). Frequency of abnormal retinal nerve fibre layer and ganglion cell layer SDOCT scans in healthy eyes and glaucoma suspects in a prospective longitudinal study. Br J Ophthalmol.

[4] Lee WJ, Na KI, Ha A, Kim YK, Jeoung JW, Park KH (2018). Combined use of retinal nerve fiber layer and ganglion cell-inner plexiform layer event-based progression analysis. Am J Ophthalmol.

[5] Lavinsky F, Wu M, Schuman JS (2018). Can macula and optic nerve head parameters detect glaucoma progression in eyes with advanced circumpapillary retinal nerve fiber layer damage?. Ophthalmology..

[6] Sung KR, Sun JH, Na JH, Lee JY, Lee Y (2012). Progression detection capability of macular thickness in advanced glaucomatous eyes. Ophthalmology..

[7] Medeiros FA, Zangwill LM, Alencar LM (2009). Detection of glaucoma progression with stratus OCT retinal nerve fiber layer, optic nerve head, and macular thickness measurements. Investig Ophthalmol Vis Sci.

[8] Wu Z, Weng DSD, Rajshekhar R, Ritch R, Hood DC (2018). Effectiveness of a qualitative approach toward evaluating OCT imaging for detecting glaucomatous damage. Transl Vis Sci Technol.

[9] Kim MJ, Park KH, Yoo BW, Jeoung JW, Kim HC, Kim DM (2015). Comparison of macular GCIPL and peripapillary RNFL deviation maps for detection of glaucomatous eye with localized RNFL defect. Acta Ophthalmol.

[10] Kim HJ, Jeoung JW, Yoo BW, Kim HC, Park KH (2017). Patterns of glaucoma progression in retinal nerve fiber and macular ganglion cell-inner plexiform layer in spectral-domain optical coherence tomography. Jpn J Ophthalmol.

[11] Tsamis E, Bommakanti NK, Sun A, Thakoor KA, De Moraes CG, Hood DC (2020). An automated method for assessing topographical structure–function agreement in abnormal glaucomatous regions. Transl Vis Sci Technol.

[12] Hood DC, De Cuir N, Blumberg DM (2016). A single wide-field OCT protocol can provide compelling information for the diagnosis of early glaucoma. Transl Vis Sci Technol.

[13] Wall M, Doyle CK, Zamba KD, Artes P, Johnson CA (2013). The repeatability of mean defect with size III and size V standard automated perimetry. Investig Ophthalmol Vis Sci.

[14] De Moraes CG, Muhammad H, Kaur K, Wang D, Ritch R, Hood DC (2018). Interindividual variations in foveal anatomy and artifacts seen on inner retinal probability maps from spectral domain OCT scans of the macula. Transl Vis Sci Technol.

[15] Leung CK, Chiu V, Weinreb RN (2011). Evaluation of retinal nerve fiber layer progression in glaucoma: a comparison between spectral-domain and time-domain optical coherence tomography. Ophthalmology..

[16] Wollstein G, Schuman JS, Price LL (2005). Optical coherence tomography longitudinal evaluation of retinal nerve fiber layer thickness in glaucoma. Arch Ophthalmol.

[17] Hollo G, Zhou Q (2016). Evaluation of retinal nerve fiber layer thickness and ganglion cell complex progression rates in healthy, ocular hypertensive, and glaucoma eyes with the avanti RTVue-XR optical coherence tomograph based on 5-year follow-up. J Glaucoma.

[18] Cheung CY, Yiu CK, Weinreb RN (2009). Effects of scan circle displacement in optical coherence tomography retinal nerve fibre layer thickness measurement: a RNFL modelling study. Eye.

[19] Asrani S, Essaid L, Alder BD, Santiago-Turla C (2014). Artifacts in spectral-domain optical coherence tomography measurements in glaucoma. JAMA Ophthalmol..

[20] Mansberger SL, Menda SA, Fortune BA, Gardiner SK, Demirel S (2017). Automated segmentation errors when using optical coherence tomography to measure retinal nerve fiber layer thickness in glaucoma. Am J Ophthalmol.

[21] Miki A, Kumoi M, Usui S (2017). Prevalence and associated factors of segmentation errors in the peripapillary retinal nerve fiber layer and macular ganglion cell complex in spectral-domain optical coherence tomography images. J Glaucoma.

[22] Vizzeri G, Bowd C, Medeiros FA, Weinreb RN, Zangwill LM (2008). Effect of improper scan alignment on retinal nerve fiber layer thickness measurements using Stratus optical coherence tomograph. J Glaucoma.

[23] Vizzeri G, Bowd C, Medeiros FA, Weinreb RN, Zangwill LM (2009). Effect of signal strength and improper alignment on the variability of stratus optical coherence tomography retinal nerve fiber layer thickness measurements. Am J Ophthalmol.

[24] Hood DC, Melchior B, Tsamis E, Liebmann JM, De Moraes CM. Did the OCT Show Progression Since the Last Visit? J Glaucoma. 2020. Publish Ahead of Print. 10.1097/IJG.0000000000001766 PMC8005430 33337725

[25] Leung CK, Cheung CY, Weinreb RN (2009). Retinal nerve fiber layer imaging with spectral-domain optical coherence tomography: a variability and diagnostic performance study. Ophthalmology..

[26] Tan BB, Natividad M, Chua KC, Yip LW (2012). Comparison of retinal nerve fiber layer measurement between 2 spectral domain OCT instruments. J Glaucoma.

[27] Mwanza JC, Chang RT, Budenz DL (2010). Reproducibility of peripapillary retinal nerve fiber layer thickness and optic nerve head parameters measured with cirrus HD-OCT in glaucomatous eyes. Investig Ophthalmol Vis Sci.

[28] Thompson AC, Jammal AA, Medeiros FA (2019). Performance of the rule of 5 for detecting glaucoma progression between visits with OCT. Ophthalmol Glaucoma..

[29] Hood DC (2017). Improving our understanding, and detection, of glaucomatous damage: an approach based upon optical coherence tomography (OCT). Prog Retin Eye Res.

[30] Hood DC, Tsamis E, Bommakanti NK (2019). Structure-function agreement is better than commonly thought in eyes with early glaucoma. Investig Ophthalmol Vis Sci.

[31] Muhammad H, Fuchs TJ, De Cuir N (2017). Hybrid deep learning on single wide-field optical coherence tomography scans accurately classifies glaucoma suspects. J Glaucoma.

[32] Thakoor KA, Li X, Tsamis E, Sajda P, Hood DC (2019). Enhancing the accuracy of glaucoma detection from OCT probability maps using convolutional neural networks. Conf Proc IEEE Eng Med Biol Soc.

[33] Asaoka R, Murata H, Hirasawa K (2019). Using deep learning and transfer learning to accurately diagnose early-onset glaucoma from macular optical coherence tomography images. Am J Ophthalmol.

[34] Yu M, Lin C, Weinreb RN, Lai G, Chiu V, Leung CK (2016). Risk of visual field progression in glaucoma patients with progressive retinal nerve fiber layer thinning: a 5-year prospective study. Ophthalmology..

[35] Kim KE, Yoo BW, Jeoung JW, Park KH (2015). Long-term reproducibility of macular ganglion cell analysis in clinically stable glaucoma patients. Investig Ophthalmol Vis Sci.

[36] Lee SH, Joiner DB, Tsamis E (2019). OCT circle scans can be used to study many eyes with advanced glaucoma. Ophthalmol Glaucoma..

