# Supplementary figures and images for: Global optical coherence tomography measures for detecting the progression of glaucoma have fundamental flaws
Source: Eye (Lond). 2021 Jan 7;35(11):2973–82. doi: 10.1038/s41433-020-01296-x (PMC8526823; doi:10.1038/s41433-020-01296-x)

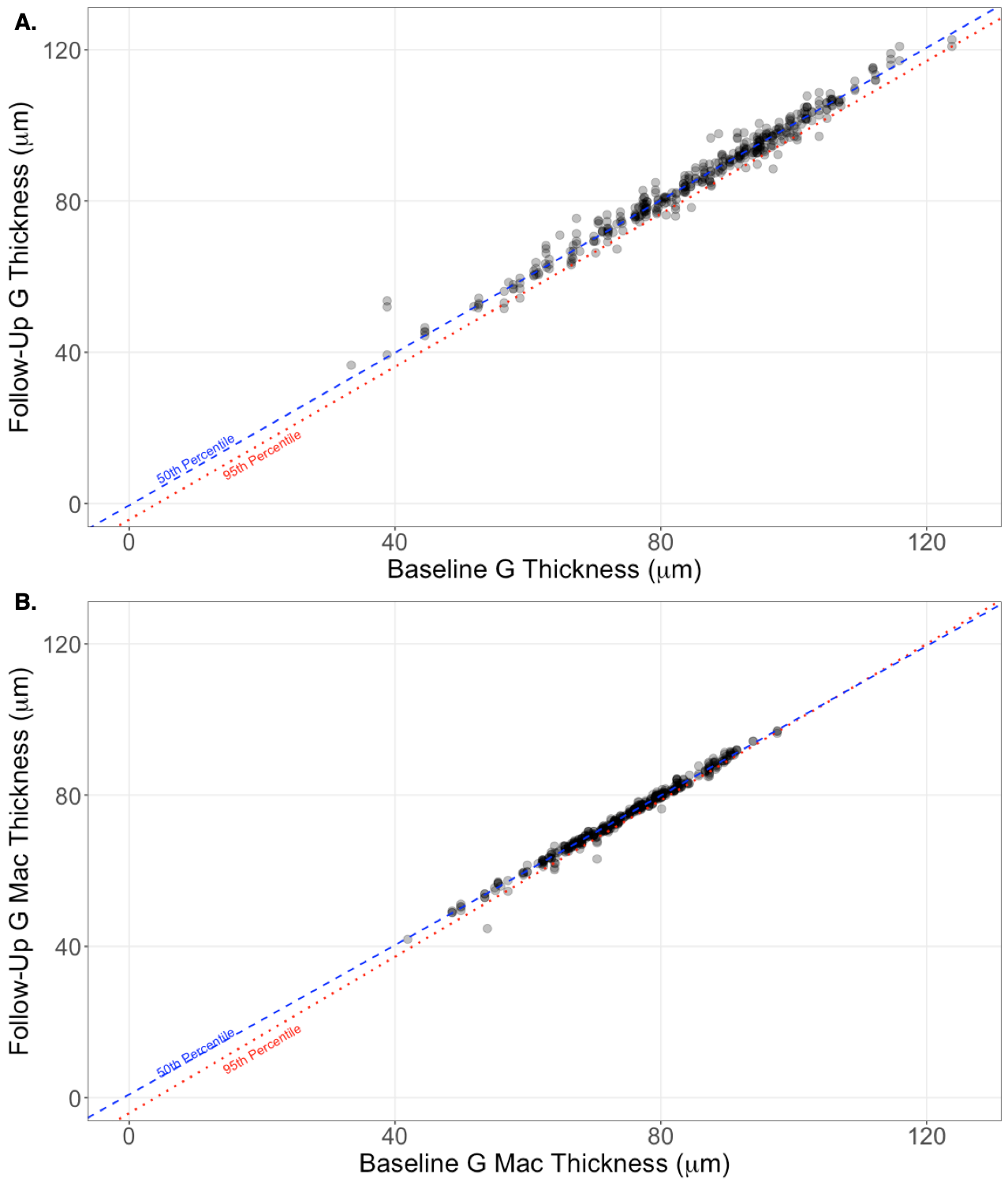

Supplement: Supplementary file 1 — Supplementary Figure 1: Quantile Regression of Baseline and Follow-Up Visits [file 41433_2020_1296_MOESM1_ESM.png]

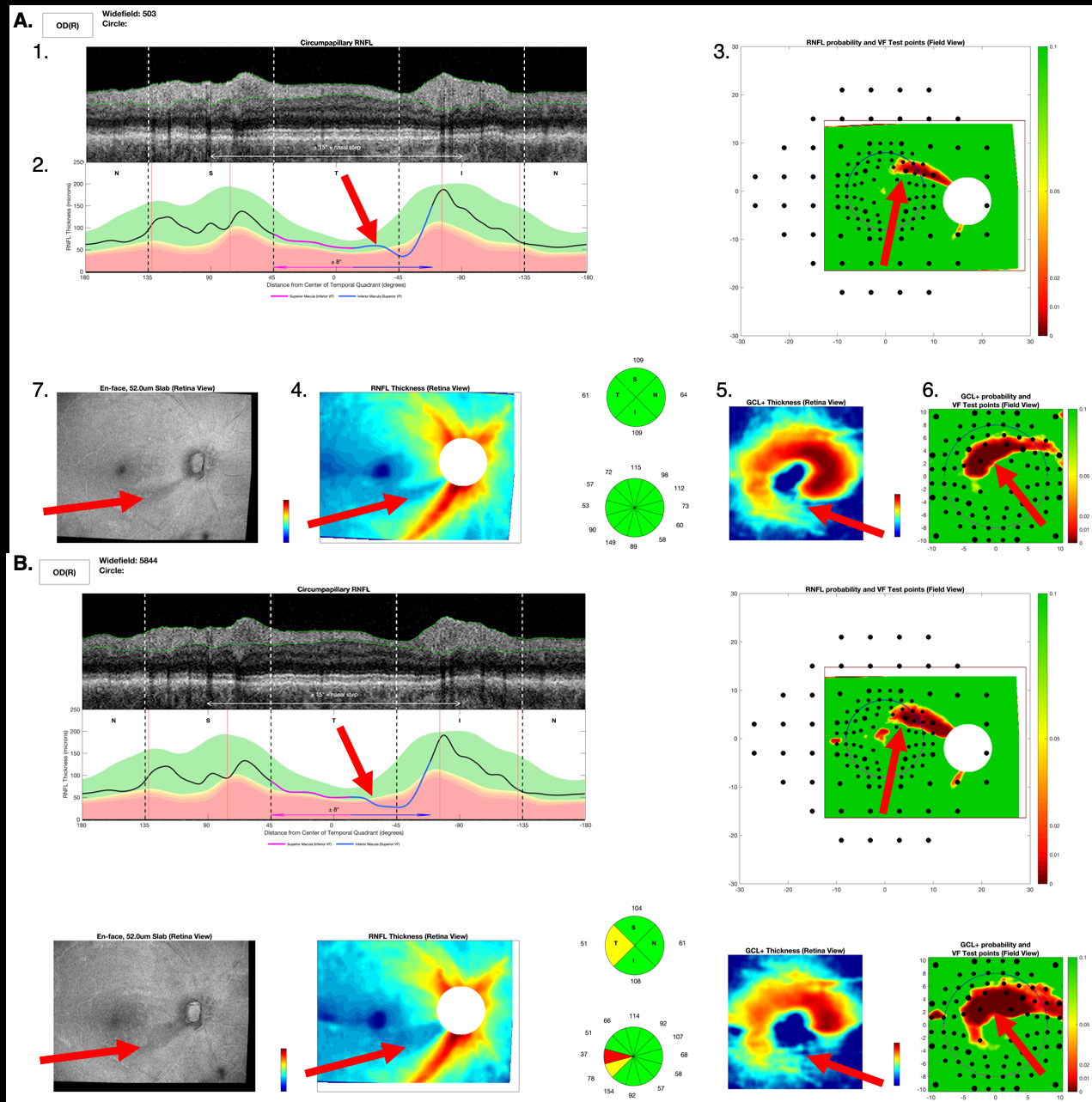

Supplement: Supplementary file 2 — Supplementary Figure 2: Example of a true positive for both metrics [file 41433_2020_1296_MOESM2_ESM.png]

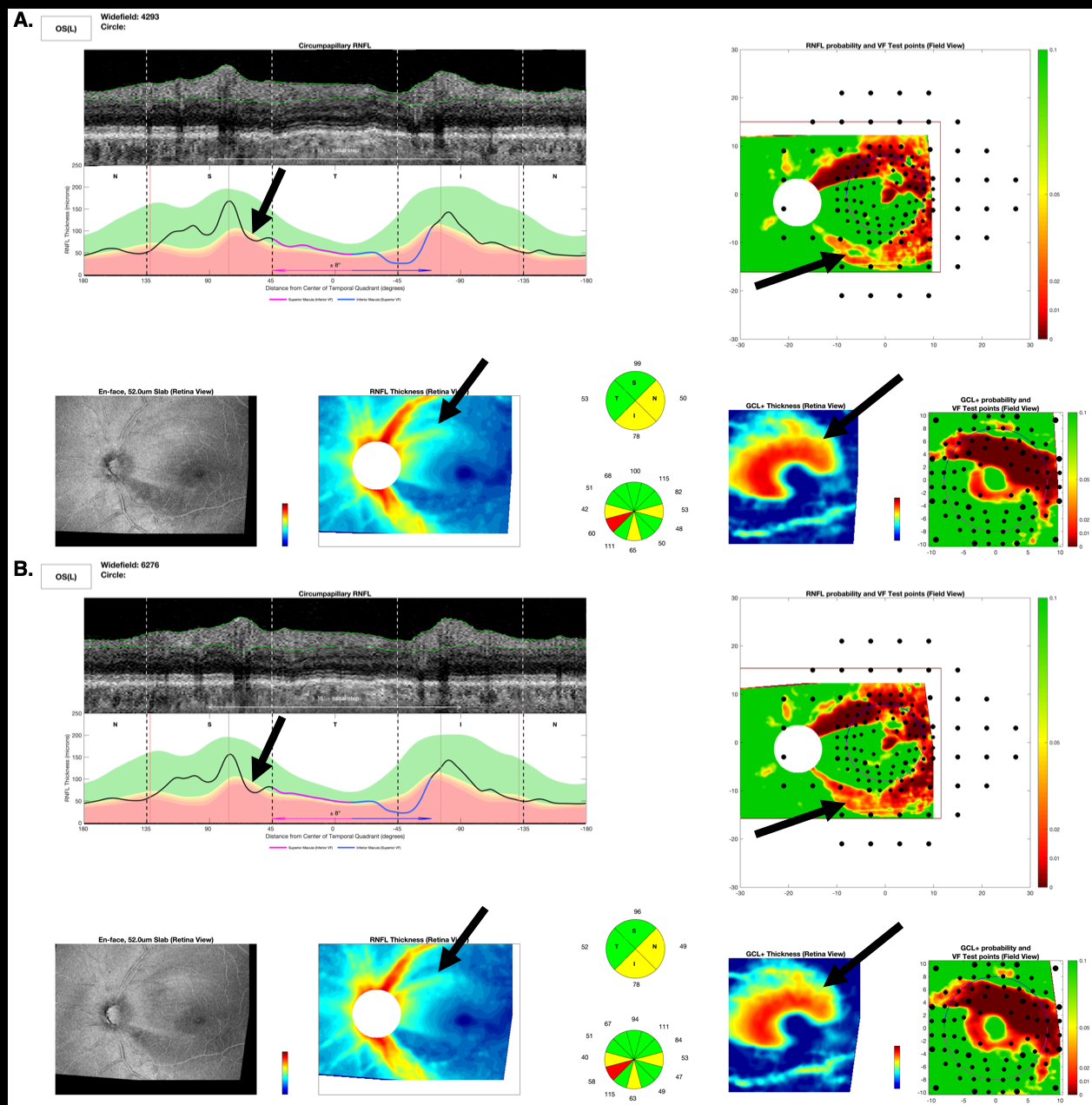

Supplement: Supplementary file 3 — Supplementary Figure 3: Example of a false negative for both metrics [file 41433_2020_1296_MOESM3_ESM.png]

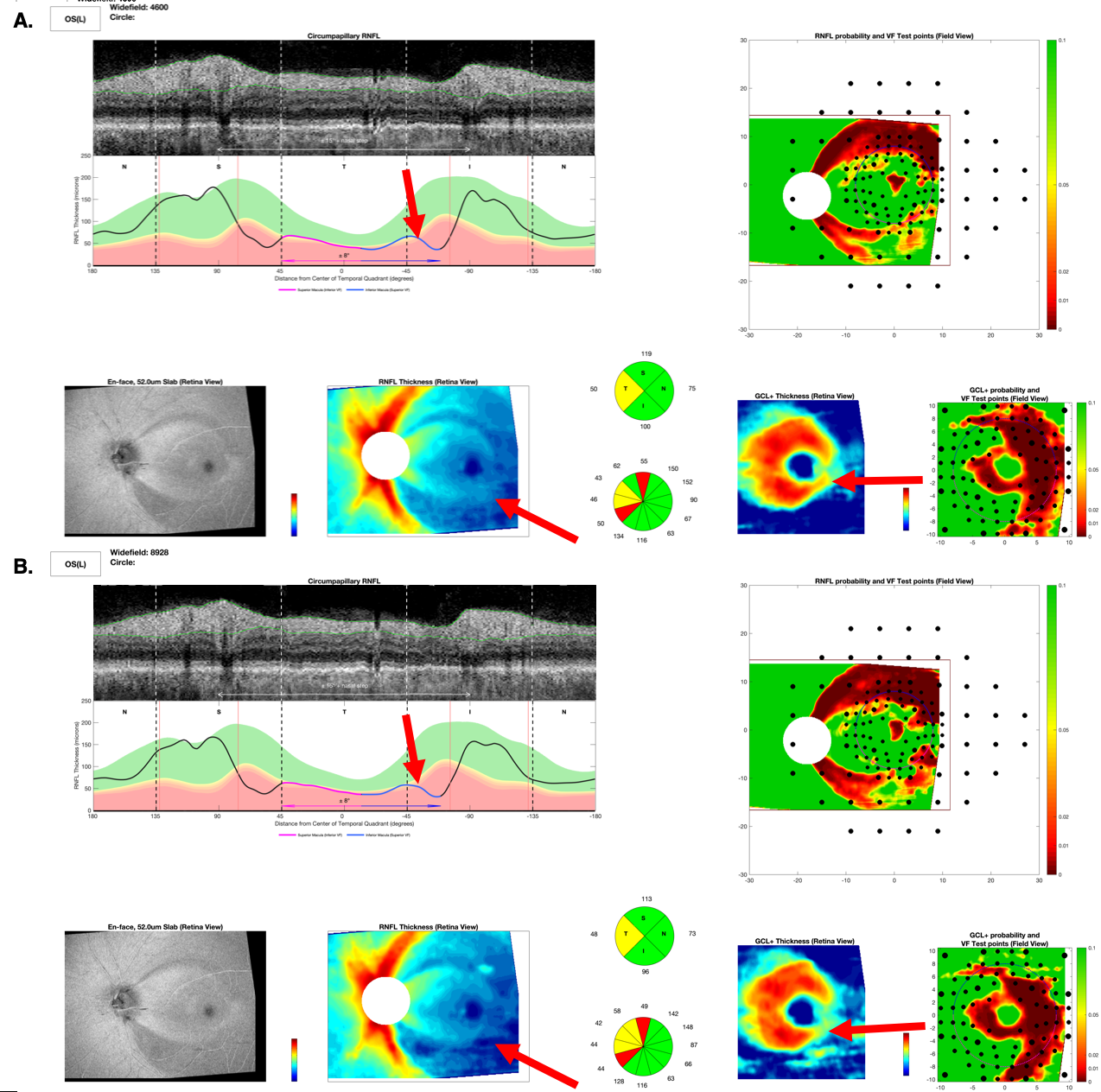

Supplement: Supplementary file 4 — Supplementary Figure 4: Example of a false negative for GONH [file 41433_2020_1296_MOESM4_ESM.png]

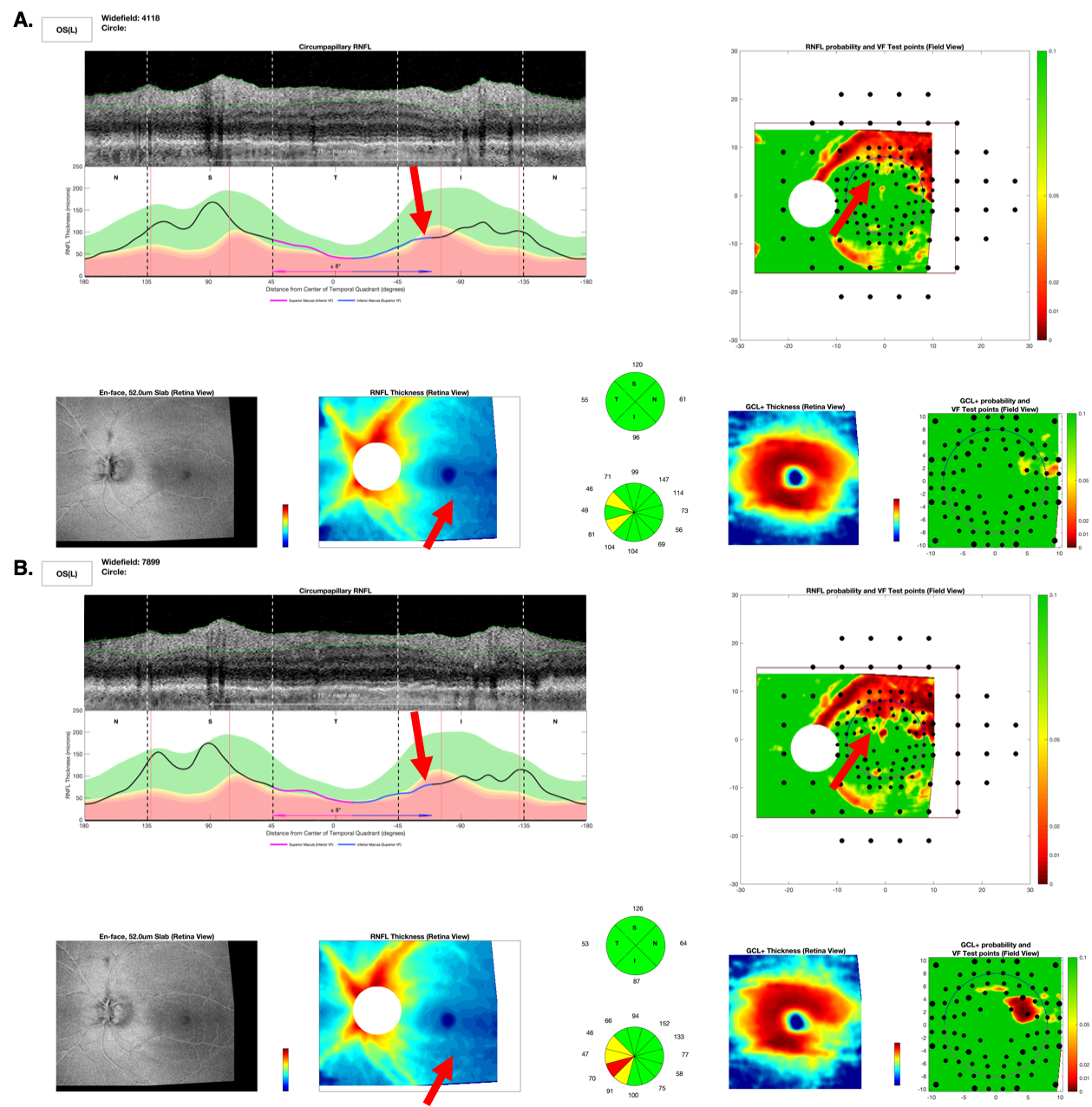

Supplement: Supplementary file 5 — Supplementary Figure 5: Example of a false negative for Gmac [file 41433_2020_1296_MOESM5_ESM.png]
